# Oral Colonization by Different *Candida* Species: First Comparative Study between Denture and Nondenture Wearers in Tunisia

**DOI:** 10.1055/s-0044-1787819

**Published:** 2024-07-23

**Authors:** Oussama Benyounes, Sana Bekri, Sameh Belgacem, Amel Labidi, Mehdi Khemis, Lamia Mansour

**Affiliations:** 1 Department of Removable Prosthodontics, Faculty of Dental Medicine, University of Monastir, Monastir, Tunisia; 2 ABCDF Laboratory of Biological, Clinical and Dento-Facial Approach, University of Monastir, Monastir, Tunisia; 3 Laboratory of Medical and Molecular Parasitology-Mycology (LP3M), Faculty of Pharmacy, University of Monastir, Monastir, Tunisia; 4 Laboratory of Microbiology, EPS Fattouma Bourguiba, Monastir, Tunisia; 5 Department of Dental Medicine, Faculty of Dental Medicine, University of Monastir, Monastir, Tunisia; 6 Research Laboratory N8 LR12SP10: Functional and Aesthetic Rehabilitation of Maxillary, University of Monastir, Monastir, Tunisia

**Keywords:** *Candida albicans*, *Candida tropicalis*, *Candida glabrata*, *Candida krusei*, removable dentures

## Abstract

**Objective** This study aimed to compare different *Candida* species present in patients with and without removable dentures to identify alterations in biofilm composition following denture wear within a Tunisian population.

**Materials and Methods** A cross-sectional study was conducted, comprising a group of patients wearing removable dentures (test group) and a control group without dentures. In the test group, two mycological samples were obtained: one from the prosthetic intaglio and another from the osteomucosal area bearing the denture. For the control group, mycological samples were collected from the oral mucosa. The collected swabs were cultured on CHROMagar *Candida* medium, and yeast counts were quantified as colony forming units (CFUs). *Candida* species were identified through chromogenic analysis.

**Statistical Analysis** The normality of quantitative variables was evaluated using the Kolmogorov–Smirnov's test. To compare means and ranks between the test and control groups, the independent samples *t* -test and the Mann–Whitney's *U* test were employed, respectively. Qualitative variables were compared using Fisher's exact test. Statistical significance was determined at a critical uncertainty value of *p* < 0.05.

**Results** A total of 150 participants were involved in this study, with 75 patients in each group. Wearing an acrylic removable denture was found to increase the number of detected *Candida* species ( *p* < 0.001) and significantly increases the overall growth of *Candida* spp. ( *p* = 0.001). Specifically, the numbers of CFUs of *Candida tropicalis* and *Candida glabrata* were elevated in denture wearers ( *p* < 0.001).

**Conclusion** Findings stemming from this study indicate that removable dentures promote the growth of *Candida* species. This can be a predisposing factor for *Candida* -associated denture stomatitis in cases of poor oral hygiene or compromised immunity. Therefore, it is imperative to emphasize the fabrication of high-quality dentures and the implementation of rigorous postdenture maintenance protocols to prevent or limit *Candida* infection.

## Introduction

*Candida* species commonly inhabit the oral cavity as commensal microorganisms in healthy individuals. Among them, *Candida albicans* is the most common species and typically remains nonpathogenic. 1 2 However, wearing removable dentures can alter the oral environment, leading to changes in the physical and biological properties of saliva and other oral structures. 3 These alterations often cause an imbalance in the local microbiota, fostering the formation of biofilm with complex structure and composition. 4 Denture wear can therefore constitute vectors of microbial virulence and act as predisposing factors to the onset of pathologies related to *Candida* spp., such as *Candida* -associated denture stomatitis (CADS). 2 5 6 7 8 9 *Candida* species contribute to localized diseases on mucosal surfaces and are associated with invasive opportunistic mycoses, constituting a major cause of nosocomial bloodstream infections. 10 Although *C. albicans* has been reported to be the principal *Candida* strain responsible for CADS, recent evidence suggests the rise of non- *albicans Candida* species as predominant pathogens in this condition. 8 11 12

The emergence of oral *Candida* species other than *C. albicans* , such as *C. krusei* , *C. glabrata* , *C. tropicalis* , and *C. parapsilosis* has garnered significant attention from research groups around the world. 3 13 14 15 16 The increase in oral *Candida* species counts following the insertion of dentures predicts the pathogenicity of these microorganisms. 12 Importantly, some of these species display resistance toward antifungal agents. 17 18

Most studies related to the identification and quantification of *Candida* species in patients wearing removable dental prosthesis, or dentures, have focused on those exhibiting symptoms of CADS, with few mycological studies exploring typical denture flora. 10 19 20 21 22

To our knowledge, this study marks the first investigation conducted in Tunisia to discern changes in mycological profiles related to *Candida* colonization among healthy wearers of removable dentures. Our primary objective was to investigate and compare the prevalence and the abundance of different *Candida* species in cohorts of Tunisian patients with and without dentures. 23

## Materials and Methods

### Ethical Approval

The study was approved by the Ethics Committee of the Faculty of Medicine of Monastir (certificate number IORG 0009738N°129/OMB 0990-0279). Before participation, all patients were informed of the study's objectives, and written consent was obtained. This study adhered to principles outlined in the Declaration of Helsinki guidelines.

### Patient Selection

This study was conducted over 3 months, from January 2023 to March 2023, at the Department of Removable Prosthodontics at the Dental Clinic of Monastir and the Mycology Laboratory at Fattouma Bourguiba University Hospital. The study included a total of 150 patients, divided into two groups: the test group and the control group. Inclusion and exclusion criteria are detailed in Table 1 .

**Table 1:** Inclusion and exclusion criteria for control and test groups

	Test group	Control group
Inclusion criteria	• Good general health as indicated by clinical record data • Wearing a uni- or bimaxillary acrylic removable dentures for at least 6 mo • Positively responding to the phone call to take part in the study	• Good general health
Exclusion criteria	• Incomplete medical history • Lack of contact number in the medical record • Being unreachable when called • Reporting during phone call that they lost or did not wear their dentures • Discovery of changes in patient's health status during the interview (e.g., diabetes, xerostomia, autoimmune diseases, HIV-positive status, undergoing chemotherapy, chronic illnesses involving immunosuppressive treatments, etc.) • Use of antifungal medications, corticosteroids, antibiotics, or antiseptic mouthwash in the previous 2 mo • Use of adhesive creams • Presence of tissue conditioners on the intaglio surface • Symptoms of CADS during clinical examination	• History of wearing removable dentures • Clinical signs of oral candidiasis or xerostomia • Use of antifungal medications, corticosteroids, antibiotics, immunosuppressants, or oral antiseptics in the previous 2 mo

Abbreviations: CADS, *Candida* -associated denture stomatitis; HIV, human immunodeficiency virus.

The test group comprised 75 patients wearing dentures who were treated at the department of prosthodontics between 2018 and 2022. Eligible participants were identified through a review of archived files, following which they were contacted by telephone and invited to participate in the test group. The control group consisted of 75 patients who consulted the department of prosthodontics for the first time for prosthetic rehabilitation.

### Data and Sample Collection

Clinical information regarding patients' current clinical condition and medical history was collected using a specifically designed clinical information sheet.

Mycological samples were taken in the morning while patients were fasting, without performing any oral or denture hygiene procedures beforehand. Sterile swabs, premoistened with a saline solution, were utilized for sample collection and then transferred to sterile tubes.

For the test group, two mycological samples were taken for each patient: one from the intaglio surfaces of the denture and another from its osteomucosal supporting area. Notably, in cases where participants were wearing bimaxillary removable dentures, the sample was taken from the upper denture.

In the control group, the mycological samples were exclusively collected from the oral mucosa surrounding the retroincisal papilla zone. This zone, covering an area of approximately 1 cm ^2^ around the retroincisive papilla, was chosen due to its accessibility and standardized anatomic alignment across all patients.

The collected swabs were transported for culture in CHROMagar *Candida* medium. After 20 to 48 hours of aerobic incubation at 35 to 37°C, yeast counts were determined and expressed as colony forming units (CFUs). Identification of *Candida* species was conducted through chromogenic analysis followed by additional biochemical testing with ID 32C biochemical identification strips (BioMérieux, France). Visual readings were conducted after 48 to 72 hours of incubation at 27°C. Species identification was finalized by analyzing sugar assimilation profiles using the APIWEB website. 24

### Operational Definitions

The obtained mycological cultures were interpreted following specified operational definitions.

- Negative culture: The culture was interpreted as negative if no yeast colonies were identified.
- Positive culture: The culture was interpreted as positive if at least one yeast colony was identified.
- Positive patient (test group): A patient in the test group was classified as positive when the oral and/or denture mycological results were positive.
- Negative patient (test group): A patient in the test group was classified as negative when both the oral and denture mycological results were negative.

### Statistical Analysis

Statistical analysis of data was performed with SPSS (Statistical Package for the Social Sciences) version 26. The normality of quantitative variables was evaluated using the Kolmogorov–Smirnov's test. Differences in means and ranks between test and control groups were compared using the independent samples *t* -test and the Mann–Whitney's *U* test, respectively. Qualitative variables were compared using Fisher's exact test. The differences observed were considered statistically significant when the critical uncertainty value was *p* < 0.05.

## Results

The study comprised a total of 150 participants. The test group consisted of 75 subjects, including 51 men and 24 women, resulting in a sex ratio of 2.12. Participants' ages ranged from 35 to 92 years, with a mean age of 60.29 ± 10.74 years. The control group consisted of 75 subjects, including 41 men and 34 women, with a sex ratio of 1.2, an age range of 35 to 81 years, and a mean age of 57.28 ± 8.74 years. Statistical analysis revealed no significant differences between the two groups regarding age and gender ( *p* > 0.05), indicating their independence for these variables.

Cultures were performed for both groups, revealing important differences in positivity rates. In the test group, 90.6% of the patients (68 out of 75) were positive, including 55 (73.33%) patients who were positive for *Candida* in both oral and denture samples, 4 (5.33%) positive only in oral samples, and 9 (12%) positive exclusively in denture samples. In the control group, 81.3% (65 out of 75) of the patient samples were positive for *Candida* .

**Fig. 1:**
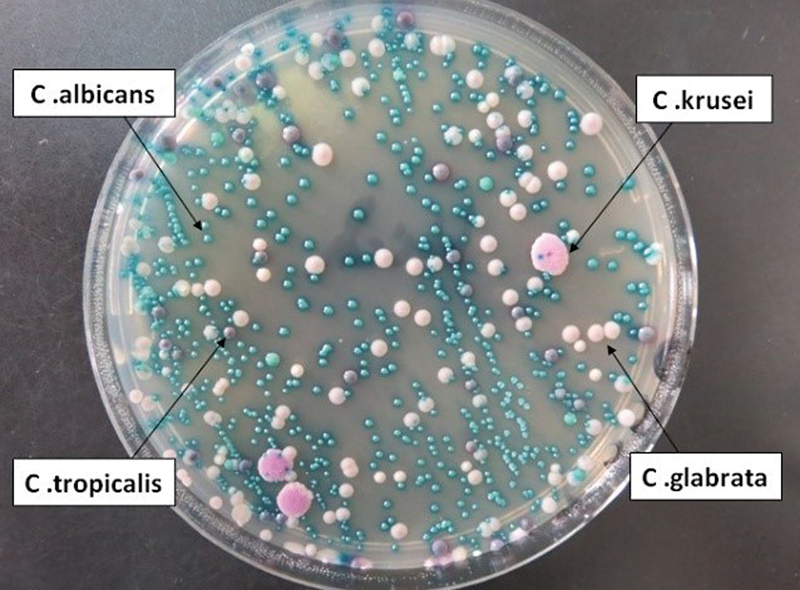
Representative mycological culture from a denture-wearing patient, demonstrating the presence of four *Candida* species.

When distinguishing the species of *Candida* detected in positive mycological samples, 72.6% of positive oral samples from the test group contained more than one *Candida* species ( Table 2 ). Further, in this group, all four *Candida* species ( *C. albicans* , *C. glabrata* , *C. krusei* , and *C. tropicalis* ) were identified in two oral samples and one denture sample ( Fig. 1 ).

**Table 2:** Distribution of *Candida* species in the positive cultures from denture-wearing and control patient samples

Group		Test		Control
Sample site		Oral *n* (%)	Denture *n* (%)	Oral *n* (%)
Number of *Candida* species detected	1 species	16 (27.1)	18 (28.1)	44 (72.13)
	2 species	20 (33.8)	20 (31.2)	13 (21.31)
	3 species	21 (35.5)	25 (39)	04 (6.56)
	4 species	2 (03.3)	1 (1.5)	0 (0)

**Fig. 2:**
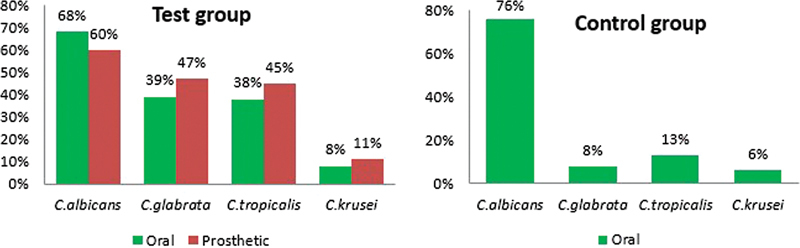
Detection frequencies of *Candida* species in positive cultures from denture-wearing and control patient samples.

A significantly greater diversity of *Candida* species was observed in the mycological samples derived from the test group as compared with the controls ( *p* < 0.001) ( Table 3 ). The detection frequencies of each *Candida* species exhibited notable differences between samples from the test and control groups ( Fig. 2 ). *Candida albicans* was the most frequently identified species in positive cultures from both the test and control groups, with detection rates of 68 and 60% for the oral and denture samples, respectively, in samples from the test group and 76% in the control samples. Importantly, non- *albicans* species including *C. tropicalis* , *C. glabrata* , and *C. krusei* displayed higher detection frequencies in the oral and denture samples from the test group as compared with the control samples ( Fig. 2 ).

**Table 3:** Comparison of the number of *Candida* species detected between denture-wearing and control patient samples

Group	*N* (%)	Median (Q1; Q3)	Minimum	Maximum	*p* -Value
a Test group	75 (50)	2 (1; 2)	0	4	
Control group	75 (50)	1 (1; 1)	0	3	<0.001 b
Total	150 (100)	1 (1; 2)	0	4	

a The maximum number of *Candida* species detected in the denture or oral cultures was used for statistical analysis.

b Significant value as determined by the Mann–Whitney's *U* test ( *p* < 0.05).

The number of CFUs in samples derived from test and control groups were compared, revealing a higher total number of *Candida* spp. CFUs in test samples ( *p* = 0.001) ( Table 4 ). Quantitative analysis of distinct species indicated that *C. tropicalis* and *C. glabrata* were notably higher in denture wearers than in nonwearers ( *p* < 0.001), while no significant differences in the abundance of *C. krusei* and *C. albicans* were noted between the test and the control groups.

**Table 4:** Quantitative comparison of *Candida* species identified denture-wearing and control patient samples

Candida species	Group	*N* (%)	Median (Q1; Q3)	Minimum	Maximum	*p* -Value
*Candida* spp. (all species)	Test	75 (50)	1,070 (5; 5,000,507)	0	505,050,005	0.001 a
	Control	75 (50)	45 (1; 1,000)	0	20,000,000	
*Candida albicans*	Test	75 (50)	22.5 (0; 50,004)	0	505,000,000	0.729
	Control	75 (50)	45 (1; 1,000)	0	10,000,000	
*Candida tropicalis*	Test	75 (50)	0.5 (0; 50,001)	0	5,050,000	<0.001 a
	Control	75 (50)	15 (0; 63)	0	100	
*Candida glabrata*	Test	75 (50)	0.5 (0; 57)	0	500,050,000	<0.001 a
	Control	75 (50)	0 (0; 0)	0	10,000,000	
*Candida krusei*	Test	75 (50)	2 (3; 42)	0	5,000,000	0.935
	Control	75 (50)	0 (0; 0)	0	100,000	

a Significant value as determined by the Mann–Whitney's *U* test ( *p* < 0.05).

## Discussion

Removable dentures are intended to replace missing teeth, fulfilling an esthetic and functional role while prioritizing biocompatibility and safety. 3 Our study shows that wearing an acrylic dentures increases the number of detected *Candida* species and significantly promotes the growth of *Candida* spp. Specifically, the number of CFUs of *C. tropicalis* and *C. glabrata* are significantly higher in denture wearers than nonwearers.

According to some reports, patients with removable dentures are at a higher risk of developing CADS due to the development of pathogenicity in oral commensal *Candida* spp. as a result of alterations to local conditions associated with denture wear. 6 20 While most of these reports predominantly focused on *C. albicans* , 25 26 few groups have evaluated the role of non *-albicans* species. 13 20 27

To the best of our knowledge, this study represents the first qualitative and quantitative mycological investigation of the different *Candida* species in denture wearers and nonwearers in Tunisia. All patients included in the study were in good general health, enabling the elimination of confounding factors that might influence the mycological microflora. Specifically, patients with weakened immune systems have a disrupted oral microflora, increasing susceptibility to oral infection. Similarly, immunosuppressive treatments and xerostomia favor elevated yeast counts and increase the risk of CADS. Consequently, patients with xerostomia or those undergoing immunosuppressive treatments were excluded from the study. 3 28 29 30

Previous studies exploring this topic have been carried out only 1 month after patients initiate denture wear. 31 32 Our study required patients in the test group to wear removable dentures for at least 6 months. This duration, as determined by Dar-Odeh and Shehabi, 27 is sufficient to detect the true effect of wearing dentures on the oral mycological microflora.

The sampling technique chosen in this study involved oral and denture swabs, while other authors utilized oral rinse samples or unstimulated saliva. 3 14 *Candida* species, particularly *C. albicans* , are known for their strong adhesive capacity and propensity to form biofilms. 33 Swab sampling of the palatal mucosa or prosthetic intaglio is therefore the optimal approach for examination as it can mechanically obtain biofilm samples. This technique also offers additional advantages in terms of simplicity, rapidity, reproducibility, and ease of transport and storage. 34

The CHROMagar *Candida* culture medium was chosen due to its selective nature and direct speciation based on color production, surpassing the performance of the commonly used Sabouraud dextrose agar. 14 35

Mycological examination revealed that 90.6% of the patients in the test group exhibited oral cultures that were positive for *Candida* spp., compared with 81.33% in the control group. In a study by Prakash et al, 13 100% of the patients wearing dentures were found to be positive for *Candida* spp., compared with 52% of patients without dentures. *Candida* spp. are commonly found in patients not wearing dentures, as they are saprophytes of the oral cavity. In the human oral microbiota, *Candida* spp. are present subclinically as commensals, and their presence is regulated by other residents of microbiota and by host immunity. 13 36 37 However, when comparing the mycological profile of the two groups, the number of *Candida* species was significantly higher in the test group than in the control samples. The oral cavity of denture wearers was found to be colonized by a mixture of *Candida* species, a trend observed in most patients in the test group.

Sant'Ana et al reported that the presence of two or more *Candida* species in the same patient could predispose the patient to recurrent stomatitis. 38 Apart from *C. albicans* , the detection frequency of the various *Candida* species was higher in denture wearers compared with patients without dentures. In our study, although *C. albicans* was the most frequently detected species in both groups, it was more frequently isolated in patients without dentures than in denture wearers. Prakash et al also reported that *C. albicans* is the predominant species (96.2%) in patients without dentures, whereas its prevalence is 58% in denture wearers. 13 This suggests a probable competition among the different *Candida* species in denture wearers that could explain the lower detection frequency of *C. albicans* in favor of other *Candida* species.

Contrary to the results of this study, Gleiznys et al concluded in their review that the prevalence of *C. albicans* is 45 to 65% in healthy individuals and that this prevalence increases to 60 to 100% in denture wearers. 6

The detection frequency of non- *albicans Candida* species was also found to be higher in the test group compared with the control group. Analysis of non- *albicans* species indicated that C. *glabrata* was most frequently isolated in denture wearers, followed by C. *tropicalis* and C. *krusei* . The frequency at which these *Candida* species are observed can have significant implications for human infections. 31

In line with our findings, Vanden et al observed a higher prevalence of *C. glabrata* (44.1%) than *C. tropicalis* (19.1%) in a group of elderly subjects with dentures. 39 Calcaterra et al also reported that the most frequently isolated non- *albicans Candida* species in the oral mucosa and dentures is *C. glabrata* , followed by *C. dubliniensis* and *C. tropicalis* . 40

While *C. glabrata* is frequently coisolated with other *Candida* species from the oral lesions, its role in the pathogenesis of CADS is still poorly understood. 41 Studies suggest that *C. glabrata* and *C. albicans* coinfection may lead to more severe symptoms and treatment complexity. 42 *Candida glabrata* can produce proteases, phospholipases, and hemolysins, and can promote biofilm formation, enabling evasion of the host immune response and generation of fungal resistance. 41 Overexpression of drug transporters and genetic mutations that modify thermotolerance are mechanisms which contribute to resistance to antifungals, such as azoles, polyenes, and echinocandins, ultimately enhancing virulence. 41

Semiquantitative analysis revealed a significant increase in the total number of *Candida* CFUs in the test group compared with the control group. Removable dentures are not inert elements; their insertion leads to changes in the physiology of the oral microbiota by inducing plaque formation that favors an abundance of potentially pathogenic bacteria and *Candida* spp. 43 44 The abundance of *Candida* in individuals wearing dentures is crucial for infection development, 20 where a higher yeast count, often exceeding 400 CFU/mL of saliva, is indicative of CADS. 45 Ghani et al reported that wearing dentures significantly enhances the pathogenic activity of oral *Candida* , leading to a mean pH decrease of 1.9 compared with 0.8 in nondenture wearers. 44 Additionally, the presence of exogenous material, particularly acrylic resin, provides new adhesion surfaces for oral biofilm. 36 Wearing dentures reduces the exposure of the osteomucosal surface to the mechanical action of saliva and the tongue, thus facilitating colonization by microbes, particularly acidogenic bacteria and *Candida* spp. 40

As shown by Du et al, 46 *Candida* spp. interact with the coexisting oral bacteria through physical attachment, extracellular signaling, and metabolic cross-feeding. These interactions enhance the cellular and biochemical composition of the biofilm, thereby increasing its pathogenic potential.

Salmanian et al demonstrated that yeast, present in the oral cavity and the digestive tract, could serve as a reservoir for *Helicobacter pylori* and play an important role in bacterial reinoculation of more distal locations in the gastrointestinal tract. 47 Consequently, diagnosing patients wearing dentures, even in the absence of symptoms, is paramount due to the importance of the oral mucosa as a primary source of bacterial reinfection.

Quantitative analysis of *Candida* species showed a significant increase in the number of CFUs of *C. tropicalis* and *C. glabrata* in the test group. Building upon this finding, the quantitative increase of these two non- *albicans Candida* species could be a predictive indicator of the development of CADS.

Multiple research groups have observed a significant increase in *C. glabrata* CFUs following the insertion of removable dentures, highlighting the species' ability to adapt to different environmental conditions. 39 48

Despite higher numbers of *C. albicans* CFUs in the test group compared with the control group, no statistically significant association was found between denture wear and *C. albicans* abundance. However, as a saprophyte of the oral cavity, *C. albicans* may enhance its pathogenicity or become an opportunistic pathogen after denture insertion. Certain strains of *C. albicans* can produce carcinogenic nitrosamines from saliva and promote neoplastic changes. 49

Wearing dentures was not found to significantly affect the number of *C. krusei* CFUs. Quantitative analyses of *C. krusei* are not frequently performed, limiting our capacity to make direct comparisons. Further, the exclusion of patients with CADS might explain the lack of association between *C. krusei* abundance and denture wear. Indeed, some reports have identified *C. krusei* as the most frequently non- *albicans Candida* species isolated from patients with CADS. 50

In their research, Samaranayake and Samaranayake. reported that *C. krusei* is less virulent than *C. albicans* in terms of its capacity to adhere to epithelial and denture surfaces. 51 Notably, *C. krusei* demonstrates distinct structural and metabolic characteristics from other *Candida* species and exhibits different behaviors in response to host defenses. Further understanding of the pathogenic potential of this yeast, together with advancements in molecular biology techniques, could further elucidate the epidemiology and the pathogenesis of *C. krusei* infections.

Denture wearers were found to be asymptomatic carriers of multiple *Candida* species more frequently than nonwearers. This underscores the potential of dentures as vectors of virulence and their role in exposing individuals to the risk of CADS. As there are no established diagnostic tests to reliably distinguish infection from colonization, the susceptibility of patients to infection could not be predicted using quantification of *Candida* CFUs alone. 52 53 Other local and systemic predisposing factors linked to host conditions, involving changes from commensal to pathogenic forms, must be considered. 8 54

Due to variations in sampling methods (swab, oral rinse, etc.) and sampling sites (tongue, oral mucosa, dentures, saliva, etc.), comparing our findings with those of other researchers constitutes a challenge. Differences in laboratory techniques and methods used to identify *Candida* species, including real-time polymerase chain reaction and diverse biochemical tests, further complicate the direct comparison of results between research groups. These discrepancies likely contribute to contradictory findings reported across different studies ( Supplementary Table S1 , available in the online version).

The proliferation and coexistence of non- *albicans Candida* species in individuals wearing removable dentures may also present a challenge in the management of candidal infections. In cases where a resistant oral candidal infection is diagnosed, it is crucial to request species identification from the laboratory, to adjust treatment strategies, including the choice of antifungal agent and dosage, accordingly. 15 55 56

Finally, although this study made efforts to quantify CFUs, the method employed was semiquantitative and therefore only provided an approximate assessment. Moreover, additional parameters that could influence oral colonization by *Candida* spp., such as the patient's age, gender, smoking habits, the duration of denture wear, and oral hygiene, were not studied. Further investigation using quantitative methods, next-generation sequencing, and controlling for confounding population variables may provide additional insight into the colonization of non- *albicans Candida* in wearers of removable dentures.

## Conclusion

Findings stemming from this study underscore the potential role of removable dentures as reservoirs for *Candida* spp. The increase in both the total number of *Candida* colonies and the variety of non- *albicans Candida* species present in samples derived from healthy denture wearers suggests an increased risk for CADS mediated by denture wear. Preventive measures should be implemented to mitigate this risk, including the provision of well-fabricated dentures and rigorous professional postdenture maintenance. Additionally, patients should be instructed on how to undertake daily denture and oral hygiene practices. Further investigation is required to delineate the exact mechanisms by which dentures alter oral *Candida* populations.

## References

[1] Bunetel L Tamanai-Shacoori Z Martin B Autier B Guiller A Bonnaure-Mallet M Interactions between oral commensal *Candida* and oral bacterial communities in immunocompromised and healthy children J Mycol Med 2019 29 03 223 232 31235209 10.1016/j.mycmed.2019.06.004

[2] Manikandan S Vinesh E Selvi D T Kannan R K Jayakumar A Dinakaran J Prevalence of *Candida* among denture wearers and nondenture wearers J Pharm Bioallied Sci 2022 14 01 S702 S705 36110628 10.4103/jpbs.jpbs_781_21 PMC9469301

[3] Bianchi C M Bianchi H A Tadano T Factors related to oral candidiasis in elderly users and non-users of removable dental prostheses Rev Inst Med Trop São Paulo 2016 58 17 27007560 10.1590/S1678-9946201658017 PMC4804554

[4] Park S E Periathamby A R Loza J C Effect of surface-charged poly(methyl methacrylate) on the adhesion of *Candida albicans* J Prosthodont 2003 12 04 249 254 15061233 10.1016/s1059-941x(03)00107-4

[5] Darwazeh A M Al-Refai S Al-Mojaiwel S Isolation of *Candida* species from the oral cavity and fingertips of complete denture wearers J Prosthet Dent 2001 86 04 420 423 11677537 10.1067/mpr.2001.118020

[6] Gleiznys A Zdanavičienė E Žilinskas J *Candida albicans* importance to denture wearers. a literature review Stomatologija 2015 17 02 54 66 26879270

[7] Zomorodian K Haghighi N N Rajaee N Assessment of *Candida* species colonization and denture-related stomatitis in complete denture wearers Med Mycol 2011 49 02 208 211 20795762 10.3109/13693786.2010.507605

[8] Salerno C Pascale M Contaldo M *Candida* -associated denture stomatitis Med Oral Patol Oral Cir Bucal 2011 16 02 e139 e143 20711156 10.4317/medoral.16.e139

[9] Bachtiar B M Fath T Widowati R Bachtiar E W Quantification and pathogenicity of *Candida albicans* in denture-wearing and nondenture-wearing elderly Eur J Dent 2020 14 03 423 428 32542630 10.1055/s-0040-1712779 PMC7440952

[10] Dağistan S Aktas A E Caglayan F Ayyildiz A Bilge M Differential diagnosis of denture-induced stomatitis, *Candida* , and their variations in patients using complete denture: a clinical and mycological study Mycoses 2009 52 03 266 271 18643887 10.1111/j.1439-0507.2008.01592.x

[11] de Freitas Fernandes F S Pereira-Cenci T da Silva W J Filho A P Straioto F G Del Bel Cury A A Efficacy of denture cleansers on *Candida* spp. biofilm formed on polyamide and polymethyl methacrylate resins J Prosthet Dent 2011 105 01 51 58 21194588 10.1016/S0022-3913(10)60192-8

[12] Abaci O Haliki-Uztan A Ozturk B Toksavul S Ulusoy M Boyacioglu H Determining *Candida* spp. incidence in denture wearers Mycopathologia 2010 169 05 365 372 20143193 10.1007/s11046-010-9275-8

[13] Prakash B Shekar M Maiti B Karunasagar I Padiyath S Prevalence of *Candida* spp. among healthy denture and nondenture wearers with respect to hygiene and age J Indian Prosthodont Soc 2015 15 01 29 32 26929483 10.4103/0972-4052.155041 PMC4762283

[14] Nayak S Kavitha B Sriram G Saraswathi T R Sivapathasundharam B Dorothy A L Comparative study of *Candida* by conventional and CHROMagar method in non-denture and denture wearers by oral rinse technique Indian J Dent Res 2012 23 04 490 497 23257483 10.4103/0970-9290.104956

[15] Zaremba M L Daniluk T Rozkiewicz D Incidence rate of *Candida* species in the oral cavity of middle-aged and elderly subjects Adv Med Sci 2006 51(1, suppl 1):233 236 17458099

[16] Vandenbussche M Swinne D Yeasts oral carriage in denture wearers Mykosen 1984 27 09 431 435 6390192 10.1111/j.1439-0507.1984.tb02056.x

[17] Cross L J Williams D W Sweeney C P Jackson M S Lewis M A Bagg J Evaluation of the recurrence of denture stomatitis and *Candida* colonization in a small group of patients who received itraconazole Oral Surg Oral Med Oral Pathol Oral Radiol Endod 2004 97 03 351 358 15024360 10.1016/j.tripleo.2003.10.006

[18] Yamin D Akanmu M H Al Mutair A Alhumaid S Rabaan A A Hajissa K Global prevalence of antifungal-resistant *Candida parapsilosis* : a systematic review and meta-analysis Trop Med Infect Dis 2022 7 08 188 36006280 10.3390/tropicalmed7080188 PMC9416642

[19] Marcos-Arias C Vicente J L Sahand I H Isolation of *Candida dubliniensis* in denture stomatitis Arch Oral Biol 2009 54 02 127 131 18950745 10.1016/j.archoralbio.2008.09.005

[20] Le Bars P Kouadio A A Bandiaky O N Le Guéhennec L de La Cochetière M F Host's immunity and *Candida* species associated with denture stomatitis: a narrative review Microorganisms 2022 10 07 143 147 35889156 10.3390/microorganisms10071437 PMC9323190

[21] Redding S W The role of yeasts other than *Candida albicans* in oropharyngeal candidiasis Curr Opin Infect Dis 2001 14 06 673 677 11964883 10.1097/00001432-200112000-00002

[22] Gauch L MR Pedrosa S S Silveira-Gomes F Esteves R A Marques-da-Silva S H Isolation of *Candida* spp. from denture-related stomatitis in Pará, Brazil Braz J Microbiol 2018 49 01 148 151 29054393 10.1016/j.bjm.2017.07.001 PMC5790581

[23] Kadir T Pisiriciler R Akyüz S Yarat A Emekli N Ipbüker A Mycological and cytological examination of oral candidal carriage in diabetic patients and non-diabetic control subjects: thorough analysis of local aetiologic and systemic factors J Oral Rehabil 2002 29 05 452 457 12028493 10.1046/j.1365-2842.2002.00837.x

[24] BioMérieux.Galeries d'identification API [Online]Available at:https://www.biomerieux.fr/diagnostic-clinique/galeries-didentification-api

[25] Nett J E Marchillo K Spiegel C A Andes D R Development and validation of an in vivo Candida albicans biofilm denture model Infect Immun 2010 78 09 3650 3659 20605982 10.1128/IAI.00480-10 PMC2937450

[26] Anuradha S Deepa K Amit T Saranjit B Candida spp in oral mucosa of denture wearers: a pilot study Natl J Integr Res Med 2017 8 06 71 74

[27] Dar-Odeh N S Shehabi A A Oral candidosis in patients with removable dentures Mycoses 2003 46(5-6):187 191 12801360 10.1046/j.1439-0507.2003.00871.x

[28] Bojang E Ghuman H Kumwenda P Hall R A Immune sensing of *Candida albicans* J Fungi (Basel) 2021 7 02 119 33562068 10.3390/jof7020119 PMC7914548

[29] Darwazeh A MG Darwazeh T A What makes oral candidiasis recurrent infection? A clinical view J Mycol 2014 2014 1 5

[30] Pasha K SA Rana M Mahanta S K Incidence of *Candida albicans* in diabetic patients with a dental prosthesis J Adv Med Dent Sci Res 2018 6 04 13 15

[31] Coulter W A Strawbridge J L Clifford T Denture induced changes in palatal plaque microflora Microb Ecol Health Dis 1990 3 77 85

[32] Muneer M Qamar K Naeem S Haroon S Candidal count in patients with complete dental prostheses Pak Oral Dent J 2011 31 01 205 207

[33] Akpan A Morgan R Oral candidiasis Postgrad Med J 2002 78 922 455 459 12185216 10.1136/pmj.78.922.455 PMC1742467

[34] Samaranayake L P MacFarlane T W Lamey P J Ferguson M M A comparison of oral rinse and imprint sampling techniques for the detection of yeast, coliform and *Staphylococcus aureus* carriage in the oral cavity J Oral Pathol 1986 15 07 386 388 3098945 10.1111/j.1600-0714.1986.tb00646.x

[35] Perry J D Freydière A M The application of chromogenic media in clinical microbiology J Appl Microbiol 2007 103 06 2046 2055 18045388 10.1111/j.1365-2672.2007.03442.x

[36] Lopes J P Lionakis M S Pathogenesis and virulence of *Candida albicans* Virulence 2022 13 01 89 121 34964702 10.1080/21505594.2021.2019950 PMC9728475

[37] Ealla K K Ghanta S B Motupalli N K Bembalgi M Madineni P K Raju P K Comparative analysis of colony counts of different species of oral streptococci in saliva of dentulous, edentulous and in those wearing partial and complete dentures J Contemp Dent Pract 2013 14 04 601 604 24309335 10.5005/jp-journals-10024-1371

[38] Sant'Ana PdeL Milan E P Martinez R Multicenter Brazilian study of oral *Candida* species isolated from AIDS patients Mem Inst Oswaldo Cruz 2002 97 02 253 257 12016452 10.1590/s0074-02762002000200019

[39] Vanden Abbeele A de Meel H Ahariz M Perraudin J-P Beyer I Courtois P Denture contamination by yeasts in the elderly Gerodontology 2008 25 04 222 228 18665849 10.1111/j.1741-2358.2007.00247.x

[40] Calcaterra R Pasquantonio G Vitali L A Occurrence of *Candida* species colonization in a population of denture-wearing immigrants Int J Immunopathol Pharmacol 2013 26 01 239 246 23527728 10.1177/039463201302600125

[41] Frías-De-León M G Hernández-Castro R Conde-Cuevas E *Candida glabrata* antifungal resistance and virulence factors, a perfect pathogenic combination Pharmaceutics 2021 13 10 152 159 34683822 10.3390/pharmaceutics13101529 PMC8538829

[42] Pärnänen P Meurman J H Samaranayake L Virtanen I Human oral keratinocyte E-cadherin degradation by *Candida albicans* and *Candida glabrata* J Oral Pathol Med 2010 39 03 275 278 20359311 10.1111/j.1600-0714.2009.00866.x

[43] Sumi Y Kagami H Ohtsuka Y Kakinoki Y Haruguchi Y Miyamoto H High correlation between the bacterial species in denture plaque and pharyngeal microflora Gerodontology 2003 20 02 84 87 14697018 10.1111/j.1741-2358.2003.00084.x

[44] Ghani F Chughtai M A Shah S A Biochemically assessed pathological activity of oral candida in denture and non denture wearers J Postgrad Med Inst 2011 25 03 188 198

[45] Mahmoudabadi A Z Drucker D B Mandall N O'brien K Johnson E M Theaker E D The oral yeast flora: effect of upper removable orthodontic appliances Microb Ecol Health Dis 2002 14 149 152

[46] Du Q Ren B Zhou X Zhang L Xu X Cross-kingdom interaction between *Candida albicans* and oral bacteria Front Microbiol 2022 13 911623 36406433 10.3389/fmicb.2022.911623 PMC9668886

[47] Salmanian A H Siavoshi F Akbari F Afshari A Malekzadeh R Yeast of the oral cavity is the reservoir of *Heliobacter pylori* J Oral Pathol Med 2008 37 06 324 328 18266659 10.1111/j.1600-0714.2007.00632.x

[48] Kaur R Domergue R Zupancic M L Cormack B P A yeast by any other name: *Candida glabrata* and its interaction with the host Curr Opin Microbiol 2005 8 04 378 384 15996895 10.1016/j.mib.2005.06.012

[49] Ramirez-Garcia A Rementeria A Aguirre-Urizar J M *Candida albicans* and cancer: can this yeast induce cancer development or progression? Crit Rev Microbiol 2016 42 02 181 193 24963692 10.3109/1040841X.2014.913004

[50] Rabelo G Noborikawa E Siqueira C Xavier da Silveira F Lotufo M Detection of single and mixed colonization of *Candida* species in patients with denture stomatitis Braz J Oral Sci 2011 10 03 184 188

[51] Samaranayake Y H Samaranayake L P *Candida krusei* : biology, epidemiology, pathogenicity and clinical manifestations of an emerging pathogen J Med Microbiol 1994 41 05 295 310 7966200 10.1099/00222615-41-5-295

[52] Lalla R V Patton L L Dongari-Bagtzoglou A Oral candidiasis: pathogenesis, clinical presentation, diagnosis and treatment strategies J Calif Dent Assoc 2013 41 04 263 268 23705242

[53] Kauffman C A Candiduria Clin Infect Dis 2005 41(41, suppl 6):S371 S376 16108001 10.1086/430918

[54] Tooyama H Matsumoto T Hayashi K *Candida* concentrations determined following concentrated oral rinse culture reflect clinical oral signs BMC Oral Health 2015 15 150 26597294 10.1186/s12903-015-0138-z PMC4657271

[55] Ramage G Tomsett K Wickes B L López-Ribot J L Redding S W Denture stomatitis: a role for *Candida* biofilms Oral Surg Oral Med Oral Pathol Oral Radiol Endod 2004 98 01 53 59 15243471 10.1016/j.tripleo.2003.04.002

[56] Lyon J P da Costa S C Totti V M Munhoz M F de Resende M A Predisposing conditions for *Candida* spp. carriage in the oral cavity of denture wearers and individuals with natural teeth Can J Microbiol 2006 52 05 462 467 16699571 10.1139/w05-148

