# Supplementary material for: Oral Colonization by Different Candida Species: First Comparative Study between Denture and Nondenture Wearers in Tunisia
Source: Eur J Dent. 2024 Jul 23;19(1):206–13. doi: 10.1055/s-0044-1787819 (PMC11750328; doi:10.1055/s-0044-1787819)
Supplement: Supplementary file 1 — Supplementary Material [file 10-1055-s-0044-1787819-s2423358.pdf]

Supplementary Table S1 An overview of the different works comparing *Candida* spp. colonization in patients with and without removable dentures

	Year of publication	City (country)	Study design	Groups of participants	Inclusion criteria	Noninclusion criteria	Sample	Laboratory techniques (culture/identification /quantification)	*Candida* species	Mains results
Bachtiar et al[9]	2020	Jakarta (Indonesia)	Comparative cross-sectional study	DW: 15<br>NDW: 15	Age ≥ 60 y<br>No smoking, no fasting, and no use of antibiotics for 3 mo before the study	Illnesses or medical problems (systemic diseases, pharmacological therapies...)	Unstimulated saliva (DW,NDW)<br>Tongue swabbing (DW,NDW)<br>Denture swabbing (DW)	Quantification of *C. albicans* by real-time polymerase chain reaction	*C. albicans*	The amount (log DNA copies) of *C. albicans* in the saliva of the DW group was significantly higher than that of the NDW group<br>The tongue dorsa samples of subjects in the NDW group showed, on average, a slightly higher count of *C. albicans* than that of the DW group (this difference was not statistically significant)
Prakash et al[13]	2015	Karnataka (India)	Comparative cross-sectional study	DW: 50<br>NDW: 50	Good general health<br>No clinical signs of oral infection including candidiasis	Use of antibiotics, antifungal, steroids, Immunosuppressive drugs in the past 6 mo	DW: swabbing tissue bearing area of the denture<br>NDW: palatal mucosa swabbing	Identification of *Candida* species by staining and biochemical tests	*C. albicans*, *C. dubliniensis*, *C. glabrata*, *C. tropicalis*	*Candida* was isolated from both the DW and NDW. Prevalence of different *Candida* species was significantly higher in DWs and it was predominated by *C. albicans* (58%), followed by *C. tropicalis* (28%), *C. dubliniensis* (12%), and *C. glabrata* (2%)
Bianchi et al[3]	2016	Mato Grosso (Brazil)	Comparative cross-sectional study	DW: 48<br>NDW: 43	Age ≥ 60 y<br>Patients wearing complete denture prostheses (applicable criterion for DW group)	Altered cognition<br>Infectious diseases (tuberculosis, leprosy)<br>Use of antibiotics or immunosuppressants frequent mouthwash users	Unstimulated saliva	*Candida* species identification using: VITEK2 Compact System equipment (BioMérieux Inc.) /manual colony counting	*Candida* spp.<br>*C. albicans*	Yeasts isolates of the genus *Candida* were obtained from (83.3%) of DW vs. (53.5%) of NDW<br>Elderly wearers of removable prostheses presented a 4.4-fold greater chance of *Candida* spp. isolation than elderly nonwearers (95% CI = 1.65–11.4)
Coulter et al[31]	1990	Belfast (Northern Ireland)	A before-and-after study	15 patients studied before the insertion of the denture and 1–4 wk after denture wearing	Good general health<br>Dental clearance for at least 1 y<br>Healthy palatal mucosa<br>Never previously worn a prosthesis	Receiving antibiotics or using antiseptic mouth rinses	Palatal mucosa swabbing	Sabouraud's dextrose agar (Oxoid) was used to culture *Candida* species<br>Counts were made based on colonial morphology on the selective plate	*Candida* spp.	*Candida* spp. were present in only a minority of samples but were detected more commonly following denture insertion and were associated with the presence of lactobacilli
Nayak et al[14]	2012	Chennai (India)	Comparative cross-sectional study	DW: 60<br>NDW: 40	DW: even patients with ill-fitting or broken dentures, who had come for replacement of the dentures<br>NDW: clinically healthy mouths<br>No systemic diseases or habits		Oral rinse technique	Isolation was done using Sabouraud's dextrose agar<br>Speciation was done using conventional methods (germ tube test, carbohydrate fermentation test, urease test) and CHROMagar method	*C. albicans*<br>*C. krusei*<br>*C. glabrata*<br>*C. tropicalis*	DW showed a statistically significant increased frequency of all *Candida* species compared with NDW ($p = 0.98$)<br>*C. glabrata* was the most common species isolated and was present in 68.3 and 37.5% of DW and

(*Continued*)

Supplementary Table S1 (*Continued*)

	Year of publication	City (country)	Study design	Groups of participants	Inclusion criteria	Noninclusion criteria	Sample	Laboratory techniques (culture/identification /quantification)	*Candida* species	Mains results
					Age ranged from 30 to 70 y in both groups					NDW, respectively<br>*C. krusei* was the least common species found and was present in 36.7 and 20% of DW and NDW, respectively
Zaremba et al[15]	2006	Bialystok (Poland)	Comparative cross-sectional study	DW: 32<br>NDW: 71	Age $\geq$ 30 y wearing complete or partial acrylic dentures for DW group		Palatal mucosa swabbing<br>Denture swabbing	Culture using Sabouraud's dextrose agar<br>Identification of the species using: API 20C AUX (bioMérieux)	*Candida* spp.<br>*C. albicans*<br>*C. krusei*<br>*C. glabrata*	Yeasts of the genus *Candida* were isolated at a comparable rate from the oral cavity of adults with and without dentures<br>A significant difference was observed only between the elderly subgroups
Muneer et al[32]	2011	Lahore (Pakistan)	A before-and-after study	40 patients newly fitted with complete dentures. Oral samples were taken at the time of denture insertion and 1 mo after wearing	Male and female with an age range of 50–65 y	History of treatment with chemotherapy or radiotherapy in the head and neck region<br>History of broad spectrum antibiotics or steroid therapy in the past 6 mo<br>Smokers and diabetics patients	Oral rinse technique	Culture on Sabouraud's dextrose agar<br>Identification Gram staining and germ tube test	*Candida* spp.	Change in candidal count was significantly higher after 1 mo of wearing complete dentures ($p = 0.0001$)
Lyon et al[56]	2006	Minas Gerais (Brazil)	Comparative cross-sectional study	DW: 112<br>NDW: 103	HIV negative<br>Never presented malignant processes and were not on any antimycotic therapy	*Candida*-associated denture stomatitis	Tongue and palatal mucosa swabbing	Culture on Sabouraud's dextrose agar<br>Identification by germ tube test	*C. albicans*<br>*C. parapsilosis*<br>*C. glabrata*<br>*C. tropicalis*<br>*C. krusei*	The major predisposing factor for the presence of *Candida* yeasts was wearing dentures ($p = 0.001$)<br>The percentage of *Candida* spp. carriers among DWs was 64.2%, while among individuals with natural teeth, this percentage was 19.4%. This difference was statistically significant ($p < 0.001$)<br>In the DW group: 65.1% of the isolates were identified as *C. albicans*, 13.7% as *C. glabrata*, 11% as *C. tropicalis*, 2.7% as *C. krusei*, 7.3% as *C. parapsilosis*

Abbreviations: CI, confidence interval; DW, denture wearers; NDW, nondenture wearers.

 © 2024. The Author(s).
